# Effects of Geometric Parameters on Mixing Efficiency and Optimization in Variable Cross Section Microchannels

**DOI:** 10.3390/mi16091001

**Published:** 2025-08-29

**Authors:** Lijun Yang, Yu Hang, Renjie Liu, Zongan Li, Ye Wu

**Affiliations:** 1 School of Mechanical Engineering, Nanjing Institute of Technology, Nanjing 211167, China; yanglj@njit.edu.cn (L.Y.); fulisayang@163.com (R.L.); 2 School of Artificial Intelligence, Nanjing Normal University of Special Education, Nanjing 210038, China; 3 School of Electrical and Automation Engineering, Nanjing Normal University, Nanjing 210046, China; chemwuye@njnu.edu.cn

**Keywords:** micromixer, geometric parameters, mixing efficiency, variable cross section

## Abstract

Micromixers are important devices used in many fields for various applications which provide high mixing efficiencies and reduce the amount of reagents and samples. In addition, effective premixing of reactants is essential for obtaining high reaction rates. In order to further improve the mixing performance, three-dimensional numerical simulations and optimizations of the flow and mixing characteristics within a variable cross section T-shaped micromixer were carried out. The effects of the geometric parameters containing channel diameter, channel shape, channel contraction and expansion ratio, and number of expansion units on the mixing were investigated with the evaluation criteria of mixing index and performance index. The optimized geometric parameters of the channel were a diameter of 0.2 mm, the shape of Sem channel, an expansion ratio of 1:3, and a number of expansion units of 7, respectively. It can be showed that the mixing efficiency of the optimized micromixer was greatly improved, and the mixing index at different velocities could reach up to more than 0.98.

## 1. Introduction

Micromixers are important devices that are widely used in many industrial and scientific fields such as chemical analysis [1], biological research [2,3], and drug delivery [4,5] for mixing liquid or gas samples of different compositions. They provide higher mixing efficiencies and reduce the amount of reagents and samples used, thereby reducing the costs and increasing the experimental efficiencies. The flow patterns of the fluids in the micromixer play an important role in the mixing efficiency and the type of the micromixer has great influence on the flow patterns. The type of the micromixer can be classified into active mixers and passive mixers according to whether they require external energy or not [6].

The external energies of active mixers are mainly provided by force fields [7,8], magnetic fields [9,10], electric fields [11,12,13], and acoustic fields [14,15]. The external driving force such as the Lorenz force and the Coulomb force of the liquids in active mixers can be significantly increased by changing the external energy, thereby shortening the mixing time and improving the mixing efficiency. Therefore, active mixers need additional energies such as magnetic or electric fields, which make the devices more complex and expensive.

Passive mixers do not require any external energy and the shapes and structures of the microchannels can be easily changed to improve the mixing efficiency. The shapes of passive mixers mainly include the snake shape [16,17], spiral shape [18,19] and tree shape [20,21] in which the fluid in the channel may accelerate due to the change in the geometry and reduce the thickness of the flow layer, thereby improving the mixing efficiency. Wang proposed a novel serpentine micromixer utilizing an ellipse curve, in which the flow direction kept continuously changing and Dean vortices were introduced throughout the whole flow path. The results suggested that the ellipse with a larger eccentricity induced stronger Dean vortices, thus better mixing performance could be obtained [16]. Khan designed and analyzed the fractal tree-like microchannels with asymmetric and symmetric bifurcation across a wide range of Reynolds numbers from 0.01 to 300. The results demonstrated that almost all the Reynolds number were better handled by the asymmetric bifurcated micromixer, because the unbalanced collision of the two fluid streams in this configuration enhanced the mixing performance [21].

The structures of passive mixers can be changed through changing the shape of the cross section [22], adding obstacles [23,24], increasing fractal structures [25,26], and adding 3D structures [27,28,29]. Shi designed six mixer models with obstacles including a square, circle, left triangle, and right triangle. They found the square obstacle mixer (SOM) was the most effective mixer, as the SOM produced significantly higher chaotic convection effects than the other three [23]. Lv proposed a micromixer with Cantor fractal baffles and evaluated the design variables including the height of the baffle (*h*), the distance of the baffle (*p*), the width of the microchannel (*L*), and the height of the microchannel on the performance of the micromixer. It can be shown that the optimized micromixer had a maximum increase of 23.75% in mixing index and the *h* and the *L* had a greater impact on the performance characteristics among all the parameters [25]. Yousefi designed micromixers with 3D twisted geometries and explored the influences of pitch number, cross section geometry, and eccentricity ratio on the performance. It can be shown that twisted micromixers with rectangular cross sections outperformed those with square cross sections by up to 34% in mixing efficiency, and the eccentricity ratio had a critical role in improving the mixing performance [29].

Passive micromixers have the advantages of energy saving, low cost, and easy integration. Therefore, improving the mixing efficiency by changing the geometric parameters of passive micromixers is a worthwhile research topic. Most of the micromixers in the studies were fabricated using photolithography, soft lithography, etching of copper, or micromilling technologies. However, there still existed some limitations in the methods, making the fabrication process complicated, expensive, and difficult. A new method of directly printing the PLA materials on the substrates to form polymer molds with variable cross sections through controlling the amount of the printing materials is introduced. The channels obtained in this method typically had some advantages, such as lower flow resistance and less low-speed zones and blind spots, which were beneficial to reduce the pressure drop and improve the mixing efficiency and performance index. When the fluids flowed into the two inlets, vortices were generated at the T-intersection. When the fluids flowed through the expansion regions, the vortices were enhanced due to convective effects. Then, the effects of the geometric parameters such as channel diameter, velocity, channel shape, channel contraction and expansion ratio, and number of expansion units on the mixing efficiency were investigated with the 3D numerical simulation.

## 2. Methods

### 2.1. Geometric Model

**Figure 1:**
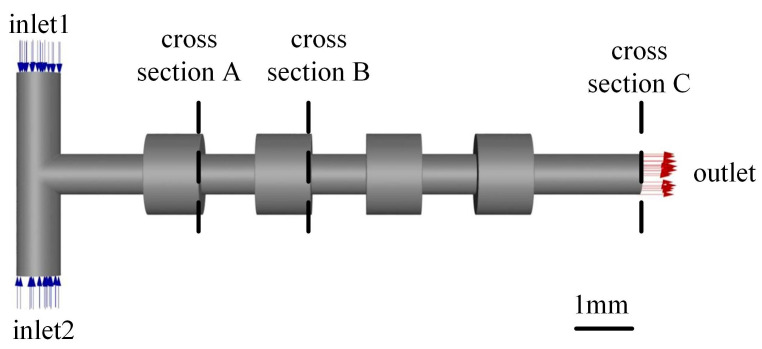
Geometric model of the micromixer.

The basic geometric model of the micromixer was a T-shaped channel with four expansion units, in which the diameter of the main channel and the diameter of the expansion units were 0.8 mm and 1.6 mm, respectively (see Figure 1). The expansion ratio was defined as the ratio of the diameter of the expansion units to the diameter of the main channel. In addition, when the diameter of the expansion units is smaller than the diameter of the main channel, the units were called contraction units and the ratio of the diameter of the contraction units to the diameter of the main channel was called the contraction ratio. The distance between the two inlets was 4 mm and the total length of the channel was 11.4 mm. The length of each expansion unit and the distance between the two neighboring units was set to be the value of 1 mm. The two cross sections intercepted at 3 mm and 7 mm from the center line of the two inlets were named cross section A and cross section B. The cross section at the outlet was named cross section C.

### 2.2. Computational Models

Since the mean free range of the internal fluid molecules in this work was much smaller than the characteristic dimensions of the equipment, it was reasonable to assume that the fluid in the flow process was a continuous medium. The following control equations could be established based on the continuous medium assumption [30].

The continuity equation is as follows:

(1) ∇⋅U=0

The momentum conservation equation is as follows:

(2) ∂U∂t+∇⋅UU=−∇P+1Re∇2U

where *U* is the velocity vector of the fluid; *P* is the pressure of the fluid on the line; and *Re* is the Reynolds number.

The convection diffusion equation is as follows:

(3) $$\frac{\partial C}{\partial t}+U\cdot \nabla C=D\nabla^{2}C$$

where *C* is the concentration of the fluid, and *D* is the diffusion coefficient of the fluid.

### 2.3. Boundary Conditions

In the numerical simulation, the initial and boundary conditions of the micromixer were set as follows: the inlet boundary type of the channel was set as a velocity inlet, and both inlets had the same velocity. The fluids in the channel were water and salt water with diffusion coefficients of 1.33 × 10^−9^ m^2^/s. The densities of water and salt water were 998.2 kg/m^3^ and 1024 kg/m^3^, respectively. The outlet boundary type of the channel was set as a pressure outlet and the gauge pressure relative to the atmosphere pressure was set to the value of 0. The flow conditions at both inlets were laminar, and there was no slippage between the fluids and the walls.

### 2.4. Evaluation Method

The standard deviation was used to describe the dispersion of the given data. We used the mixing index to describe the mixing effects of the fluids in the microchannel (as shown in Equations (4) and (5)), where *σ* was the standard deviation of the concentration or mass fraction, *N* was the number of nodes on the cross section being counted, *C*_i_ was the concentration or mass fraction at each point on the cross section being counted, ${\bar{C}}_{m}$ was the expected value of the concentration or mass fraction on the cross section being counted (*C_m_* = 0.5), *σ*^2^_max_ was the maximum variance of the concentration or mass fraction, and *M* was the mixing index. If the two materials could not be mixed at all, then *M* = 0; if the two materials could be mixed completely, then *M* = 1. In addition, the fluids could be considered to be completely mixed when *M* > 0.95. Therefore, the fluids in the channel were considered to be completely mixed when *M* > 0.95.

(4) $$\sigma =\sqrt{\frac{1}{N}}\Sigma {\left(C_{i}-{\bar{C}}_{m}\right)}^{2}$$

(5) $$\sigma_{\max}^{2}={\bar{C}}_{m}\left(1-{\bar{C}}_{m}\right)$$

(6) $$M=1-\sqrt{\frac{\sigma^{2}}{\sigma_{\max}^{2}}}$$

However, when the materials were mixed in microchannels, the mixing index and the pressure drop could not be taken as determinants of the mixing efficiency independently. Instead, the mixing index and pressure drop should be taken into consideration together. Therefore, in order to describe the performance of the micromixers, the performance index (*PI*) was introduced to carry out the evaluation, as is shown in Equation (7) which follows, in which Δ*p* was the pressure drop between the inlet and outlet of the mixer:

(7) $$PI=\frac{M}{\Delta p}$$

### 2.5. Grid Independence Verification and Validation of Validity

In numerical simulation, the choice of mesh structure had a significant influence on the accuracy of the simulation. So, the grid independence verification was a necessary step to ensure the rationality and accuracy of the obtained solutions. In grid independence verification, numerical calculations with different sizes of meshes were performed to evaluate whether the simulation tends to be stable. After that, we could confirm that the selected mesh could adequately capture the physical phenomena and avoid the deviation of the results due to improper mesh settings.

**Figure 2:**
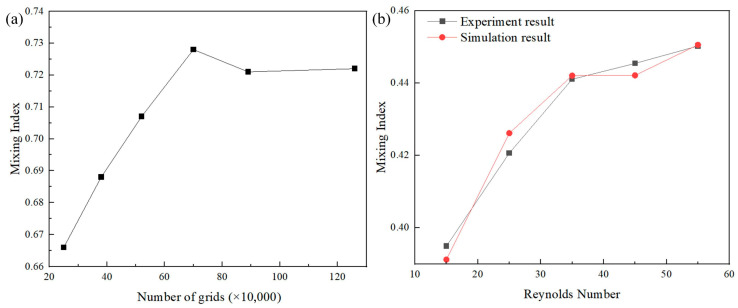
(**a**) The relationship between the number of the grids and the mixing index, and (**b**) the relationship between the Reynolds number and the mixing index of the experiment result and the simulation result.

In order to verify the grid independence, a stepwise refinement method was used to compare the calculation results under different mesh densities. Six grid systems of 250,000, 380,000, 520,000, 700,000, 890,000, and 1,260,000 grids were designed for numerical simulations, respectively. The calculation results are shown in Figure 2a. The maximum error between 890,000 and 1,260,000 grids was only 0.14%. The grid system of 890,000 grids was selected for the calculation of the micromixer of this model. Other models also followed the same method for the selection of the number of grids.

In order to verify the validity of the numerical simulation method, the simulation was performed and the result was compared with the experiment result [31]. As shown in Figure 2b, the simulation result was basically consistent with the experiment result, and the maximum change in the mixing index was only 1.31% when the Reynolds number was 25. Therefore, the boundary conditions, grid number selections, and simulation results used in our model were reasonable and effective.

## 3. Results and Discussion

### 3.1. Effects of Channel Diameter and Velocity on Mixing

**Figure 3:**
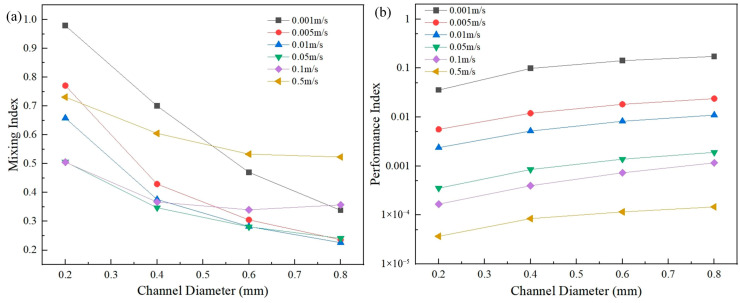
(**a**) The relationship between the channel diameter and the velocity and the mixing index; (**b**) the relationship between the channel diameter and the velocity and the performance index.

Different velocities and different channel diameters will result in different flow times and lateral diffusion times of the fluids in the channel. Therefore, the inlet velocity and channel diameter will have some effects on the mixing process of the fluids in the channel. The effects of channel diameters and inlet velocities on mixing are shown in Figure 3, in which the channel diameters and the inlet velocities are in the range of 0.2 mm–0.8 mm and 0.001 m/s–0.5 m/s, respectively.

The diameter and the velocity had great influences on the mixing index and the performance index (see Figure 3). The channel with a diameter of 0.2 mm had the best mixing index at all velocities and the samples were almost completely mixed at a velocity of 0.001 m/s. However, a smaller diameter led to an excessive pressure drop in the microchannels, which resulted in a lower performance index. On the contrary, the channel with a diameter of 0.8 mm had a poor mixing effect, but the performance index was the best due to the smallest pressure drop.

Figure 3 shows that in the case of smaller channels, the smaller the inlet velocity, the higher the mixing index. This is because the fluids stayed in the channel for a longer time at low velocities and made better use of intermolecular diffusion for mixing. However, in the case of larger channels, the channel with a velocity of 0.5 m/s had a higher mixing index than that of 0.001 m/s. This is due to the fact that the convection effects dominated the mixing process of the fluids in the larger channel at a larger velocity.

**Figure 4:**
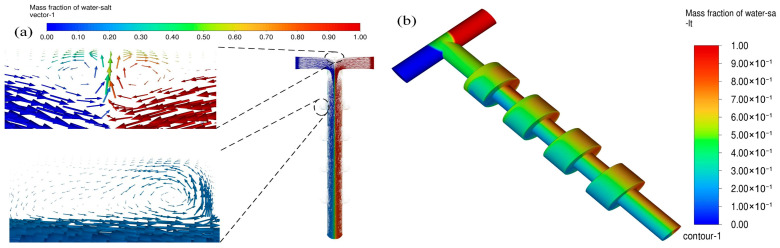
(**a**) Vector plot of salt water mass fraction at the velocity of 0.5 m/s; (**b**) cloud plot of salt water mass fraction at the velocity of 0.5 m/s.

Figure 4 shows the vector plot and cloud plot of salt water mass fraction at a velocity of 0.5 m/s. As is shown in Figure 4a, the two fluids generated vortices at the T-shaped intersection region and the sudden expansion regions, and a secondary vortex was even produced at the sudden expansion regions. The reason for this may be that the convection effects occupied a dominant role in the material mixing process at a speed of 0.5 m/s, which broke the laminar flow state and enhanced the convective diffusion.

**Figure 5:**
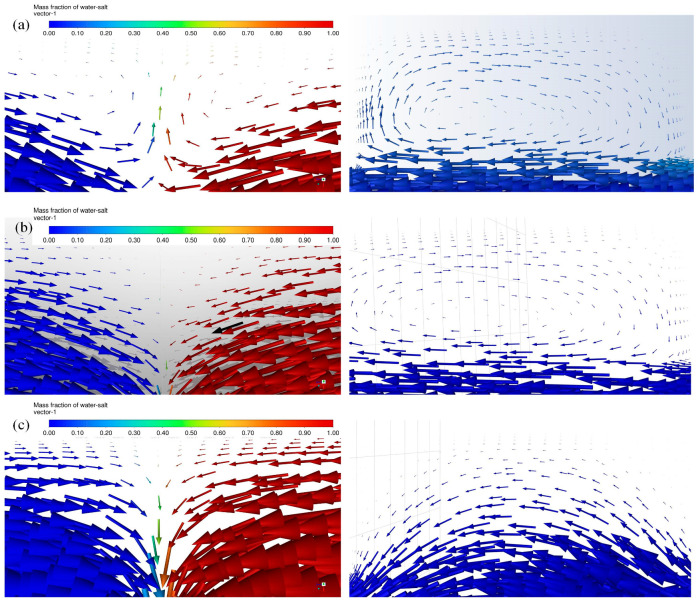
(**a**) Vector plot of salt water mass fraction at the T intersection region and the expansion region at 0.1 m/s; (**b**) vector plot of salt water mass fraction at the T intersection region and the expansion region at 0.05 m/s; (**c**) vector plot of salt water mass fraction at the T intersection region and the expansion region at 0.001 m/s.

Figure 5 shows the vector plots of salt water mass fraction at velocities of 0.1 m/s, 0.05 m/s, and 0.001 m/s, respectively. As shown in Figure 5a,b, when velocity decreased to 0.1 m/s and 0.05 m/s, the vortices at the T-shaped intersection and the sudden expansion positions were clear, but the secondary vortex disappeared which decreased the mixing index. This is because the convection effects weakened when velocity decreased. As is shown in Figure 5c, when the velocity continued to decrease to 0.001 m/s, the vortex almost disappeared. In this case, molecular diffusion occupied the dominant role in the material mixing process and diffusion time was extended greatly, which improved the mixing index.

### 3.2. Effects of Channel Shape on Mixing

The mixing index of materials in the microchannels was influenced by the shape of the channels. We compared the straight (Str), square (Squ), and semicircular (Sem) microchannels with a channel diameter of 0.2 mm. We selected three characteristic velocities for material mixing: a diffusion-dominant velocity of 0.001 m/s, a velocity of 0.05 m/s in which both diffusion and convection play significant roles, and a convection-dominant velocity of 0.5 m/s.

**Figure 6:**
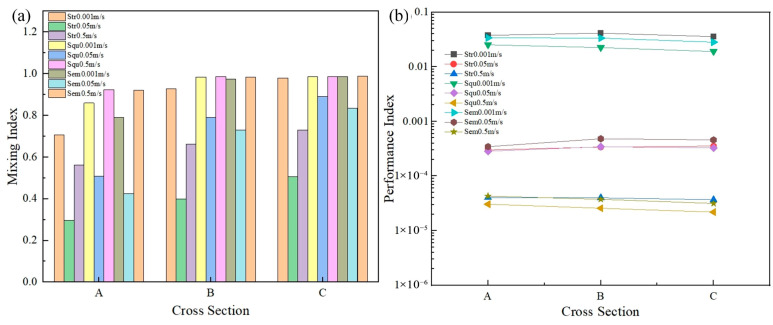
(**a**) Relationship between the channel shapes and the mixing index at cross sections A, B, and C; (**b**) relationship between the channel shapes and the performance index on cross sections A, B, and C.

The simulation results are shown in Figure 6. It can be seen that the mixing indices of the Squ channel and the Sem channel were more effective than those of the Str channel at all three cross sections with various velocities. When velocity was 0.5 m/s, the convection effect was significantly enhanced due to the change in the channel shape, and the mixing indices of the Squ channel and Sem channel were higher than those of the Str channel at three cross sections by 65.06% and 64.17%, 48.87% and 48.57%, and 35.05% and 35.09%, respectively.

**Figure 7:**
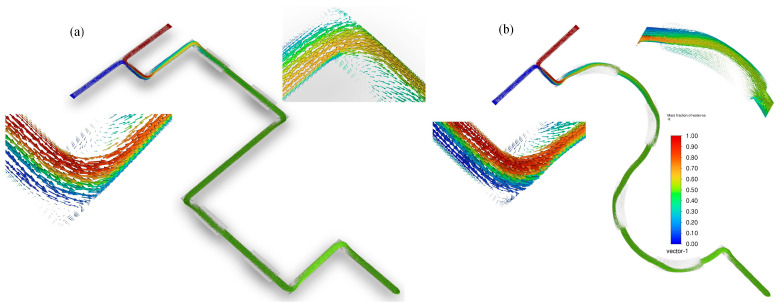
(**a**) Vector plot of salt water mass fraction in Squ channel at 0.5 m/s; (**b**) vector plot of salt water mass fraction in Sem channel at 0.5 m/s.

It can be seen that vortices were generated in the Squ channel at each turn, and the vortices at the sudden expansion positions were almost identical to the Str vortices, which means the Squ channel generated six more vortexes than the Str channel and significantly promoted the mixing efficiency of the materials (see Figure 7a). However, the overall path of the Squ channel was longer, which led to a larger pressure drop and a lower *PI* value (see Figure 6b).

**Figure 8:**
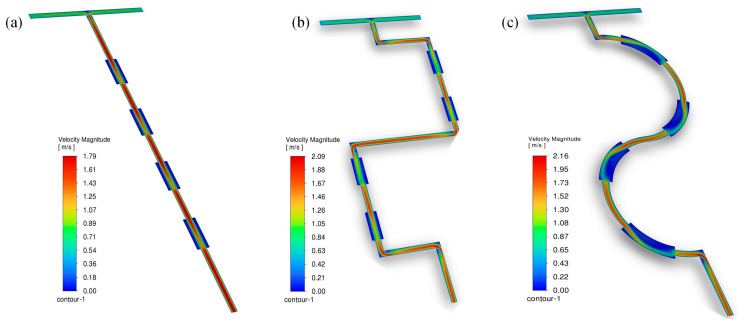
(**a**) Velocity clouds of the fluids in Str channel; (**b**) velocity clouds of the fluids in Squ channel; (**c**) velocity clouds of the fluids in Sem channel.

Compared with the Str channel, the Sem channel had a smaller range of vortices in the turn (see Figure 7b). The reason may be that during the flow process, most of the materials flowed along the center line of the channel at a fast speed due to inertial force and other materials would flow to the wall at a slow speed and gather on the side at the sudden expansion positions (see Figure 8). The materials did not have enough time to be mixed, and the return flow and the vortex were generated, which greatly improved the mixing. Compared to the Str channel, the Sem channel had a longer overall path which produced a larger pressure drop. However, compared to the Squ channel, the flow path was relatively gentle. The pressure drop was much smaller than the Squ channel (see Figure 6b).

As a result, the mixing index of the Sem channel was much higher than that of the Str channel, which was almost the same as that of the Squ channel, and the pressure drop was smaller than that of the Squ channel, which had a higher performance index. Therefore, the Sem shape was selected as the best shape of the channel.

### 3.3. Effects of Channel Contraction Ratio and Expansion Ratio on Mixing

**Figure 9:**
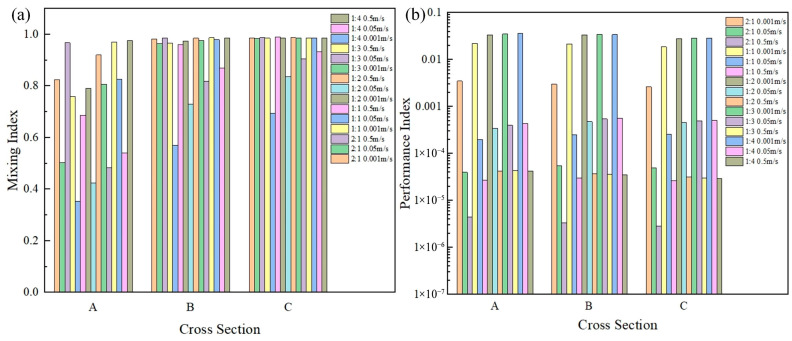
(**a**) Relationship between the contraction ratio and expansion ratio and the mixing index on the cross sections A, B, and C; (**b**) relationship between the contraction ratio and expansion ratio and the performance index on the cross sections A, B, and C.

The effects of the channel contraction ratio and expansion ratio on mixing were researched in which the ratios were 2:1, 1:1, 1:2, 1:3, and 1:4, respectively, and the results are shown in Figure 9. It can be noted that the channels with a contraction ratio of 2:1 and expansion ratios of 1:2, 1:3, and 1:4 were favorable for the mixing of the materials compared to the uniform channel with the ratio of 1:1 at all the three velocities.

When the channel contracted with a ratio of 2:1 (see Figure 9b), the contraction resulted in a significant pressure drop, which decreased the performance index. However, when the channel expanded with the ratios of 1:2, 1:3, and 1:4, not only did the mixing index significantly improve, but the pressure drops also decreased which improved the performance index. When the velocity was 0.001 m/s, the mixing indices of the channels with the expansion ratios of 1:2, 1:3, and 1:4 were improved by 0.08%, 0.09%, and 0.08% compared to those of the uniform channel with the ratio of 1:1 at the outlet. The performance indices were improved by 50.53%, 53.72%, and 53.19% at the outlet, respectively. In addition, when the velocity was 0.05 m/s, the mixing indices of the channels with expansion ratios of 1:2, 1:3, and 1:4 were 20.43%, 30.39%, and 34.5% higher than those of the uniform channel with the ratio of 1:1 at the outlet, and the performance indices were 82.9%, 93.31%, and 96.54% higher, respectively. Furthermore, when the velocity was 0.5 m/s, the mixing indices of the channels with expansion ratios of 1:2, 1:3, and 1:4 were almost the same as those of the uniform channel with the ratio of 1:1 at the outlet, but the performance indices were 19.08%, 14.05%, and 10.96% higher, respectively. Therefore, microchannels with the expansion ratio of 1:3 were selected.

### 3.4. Effects of the Number of Expansion Units on Mixing

**Figure 10:**
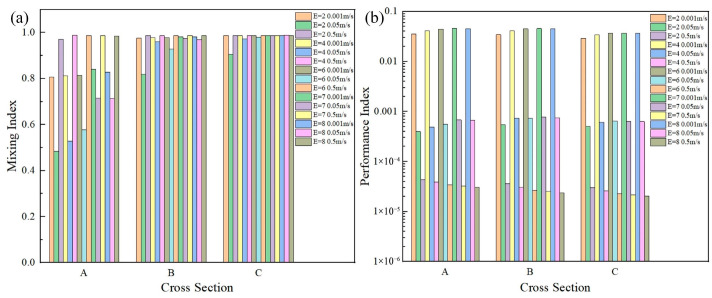
(**a**) The relationship between the *E* and the mixing index on the cross sections A, B, and C; (**b**) the relationship between the *E* and the performance index on the cross sections A, B, and C.

**Figure 11:**
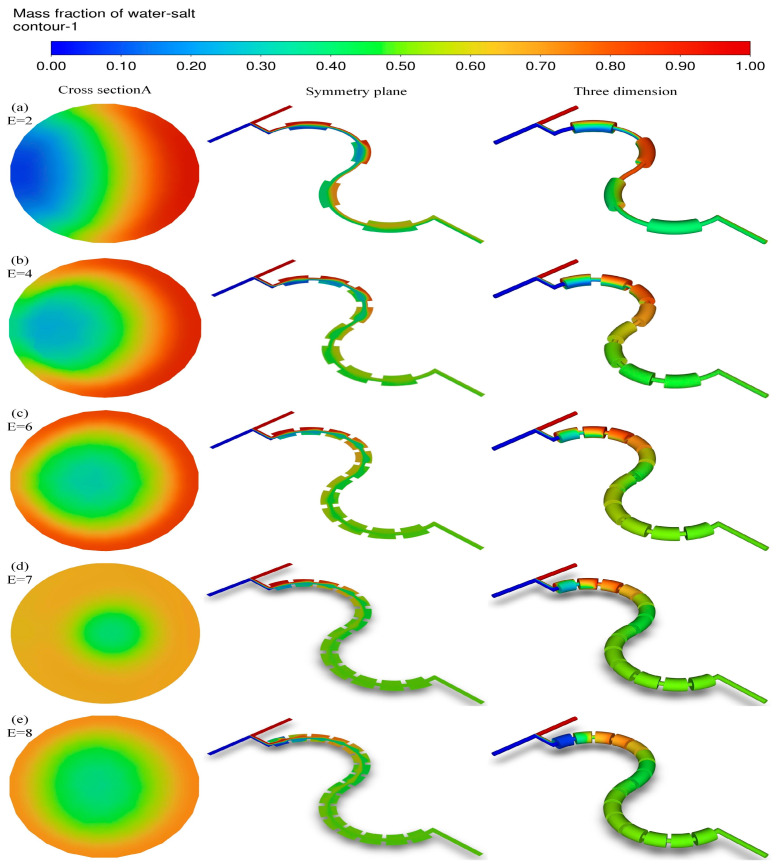
(**a**) Cloud plots of salt water mass fraction in the channel with an *E* of 2; (**b**) cloud plots of salt water mass fraction in the channel with an *E* of 4; (**c**) cloud plots of salt water mass fraction in the channel with an *E* of 6; (**d**) cloud plots of salt water mass fraction in the channel with an *E* of 7; (**e**) cloud plots of salt water mass fraction in the channel with an *E* of 8.

The vortices were created at the expansion region of each unit, which led to the fluids making better use of the convection effect. Therefore, creating more expansion units could improve the mixing efficiency. The effects of the number of expansion units on mixing were researched, in which the number of the units (*E*) were 2, 4, 6, 7, and 8, respectively, (see Figure 10 and Figure 11).

It can be seen from Figure 10a and Figure 11 that at cross section A the channels with an *E* of 4, 6, 7, and 8 had higher mixing indexes at all three velocities than those of the channel with an *E* of 2. Especially at a velocity of 0.05 m/s, the mixing indices were higher by 9.09%, 19.21%, 47.52%, and 47.31%, and the performance indices were higher by 22.02%, 38.79%, 69.36%, and 67.13%, respectively. The reason may be that an increase in *E* could make the fluids change from molecular diffusion to convective mixing, which improved the mixing efficiency. However, these positive effects were no longer significant when the number of the expansion units exceeded 7 and the performance index changed little. It should be noted that the number of the expansion units was considered to be 7.

When velocity was 0.001 m/s and 0.05 m/s, an increase in *E* could improve the mixing index and performance index (see Figure 10). However, when velocity was 0.5 m/s, the performance index decreased when *E* increased at a velocity of 0.5 m/s. This is because the flows might be more unstable at a velocity of 0.5 m/s, and an increase in *E* caused phenomena such as flow separation or additional vortices which resulted in a relatively larger pressure drop, further leading to a lower performance index.

It can be seen that our work provided a new micromixer with high mixing efficiency. Future work could be focused on nanotechnology [32] and advanced near-infrared imaging [33,34], where nanochannels can be made and characterized.

## 4. Conclusions

We have proposed a new passive micromixer with variable cross sections through changing the geometric parameters containing the channel diameter, channel shape, channel contraction, expansion ratio, and number of expansion units. The micromixer structure was continuously optimized through a three-dimensional numerical simulation with the evaluation criteria of mixing index and performance index. The optimized channel diameter was 0.2 mm as it had the best mixing index at all velocities and high performance index, although it caused a large pressure drop. The optimized channel shape was the Sem channel, because the mixing index was much higher than that of the Str channel and the pressure drop was smaller than that of the Squ channel. The sudden expansion of the variable cross section generated vortices and reduced the pressure drop, which led to a high mixing index and high performance index. It can be noted that a gain in the sudden expansion ratio of more than 1:3 was no longer obvious. It can be generated that the optimized channel shape was 1:3. The increase in the number of expansion units could make the fluids change from molecular diffusion to convective mixing, which improved the mixing efficiency. The positive effects were no longer significant when number of expansion units exceeded 7; therefore, the optimized number of expansion units was 7. The mixing efficiency of the optimized micromixer was greatly improved. The mixing index at different velocities could reach up to more than 0.98.

## Data Availability

The original contributions presented in this study are included in the article. Further inquiries can be directed to the corresponding authors.
